# Supplementary material for: Immersive virtual reality simulation for undergraduate nursing students: Enhancing mental health care for migrants - A mixed method study protocol
Source: PLoS One. 2025 Sep 25;20(9):e0333434. doi: 10.1371/journal.pone.0333434 (PMC12463210; doi:10.1371/journal.pone.0333434)
Supplement: Attachment — Submitted filename: Response to reviewers.docx [file pone.0333434.s002.docx]

Editor Comments: Dear Authors, Thank you for submitting your manuscript, "Immersive Virtual Reality Simulation for Undergraduate Nursing Students: Enhancing Mental Health Care for Migrants - A Mixed Method Study Protocol," to Plos One. While we appreciate the innovative approach of utilizing immersive virtual reality (IVR) to address a critical gap in nursing education, particularly in the context of migrant mental health care, we have identified some areas that require further attention before the manuscript can be considered for publication. Specifically, we require greater detail on the development procedure of the IVR intervention. A more thorough description of the design and creation process is needed, to include specific scenarios and content, the theoretical basis for the simulation design, technical aspects, and insights from pilot testing and refinement processes.

- Additional information has now been added to the Phase 1 - Development of the Intervention

In addition, while the manuscript mentions that an integrative review will inform the simulation design, it requires a more robust explanation of how these findings translate into specific elements of the IVR experience.

- Additional information has now been added to the Phase 1 - Development of the Intervention

Moreover, we encourage you to consider reviewers points carefully and revise your manuscript accordingly. We look forward to receiving a revised version that addresses these concerns.

- Thank you!

Reviewers' comments:

Have the authors described where all data underlying the findings will be made available when the study is complete? The [PLOS Data policy](https://can01.safelinks.protection.outlook.com/?url=https%3A%2F%2Ftrack.editorialmanager.com%2FCL0%2Fhttps%3A%252F%252Fjournals.plos.org%252Fplosone%252Fs%252Fmaterials-and-software-sharing%2F1%2F010f0197e51f25a6-39664cc5-2cf9-48c6-9012-e2bbf9f72271-000000%2FPlU-EPZWAbOKeNpt-ydTqFqVkIPExEmumkaYAMziRPw%3D218&data=05%7C02%7Cg.barbo%40usask.ca%7Cd00aab2d3245456b171b08ddbd5c08da%7C24ab6cd0487e47229bc3da9c4232776c%7C0%7C0%7C638874925193335981%7CUnknown%7CTWFpbGZsb3d8eyJFbXB0eU1hcGkiOnRydWUsIlYiOiIwLjAuMDAwMCIsIlAiOiJXaW4zMiIsIkFOIjoiTWFpbCIsIldUIjoyfQ%3D%3D%7C0%7C%7C%7C&sdata=1ksmoHH0sUWvK2gSRVAbrLeTALMDwA7sUOMAjozcKn4%3D&reserved=0) requires authors to make all data underlying the findings described in their manuscript fully available without restriction, with rare exception, at the time of publication. The data should be provided as part of the manuscript or its supporting information, or deposited to a public repository. For example, in addition to summary statistics, the data points behind means, medians and variance measures should be available. If there are restrictions on publicly sharing data—e.g. participant privacy or use of data from a third party—those must be specified. Reviewer #2: No

- This concern has been addressed and clarified in the Ethical Considerations section

Reviewer #1: Ethical Considerations and Participant Protection: The protocol states that ethics approval has been obtained from the University of Saskatchewan and McGill University. As the study is in phase 2, it would be helpful to include the ethics approval number and date of approval.

- Ethics approval numbers and dates of approval has been specified at the Status and Timeline section

As the participants were nursing students, further clarification is needed regarding how the risk of indirect coercion was avoided, especially if the researcher was their faculty member. Was recruitment conducted by a party not directly involved in the teaching process?

- Explanation has been added to the Ethical Considerations section

Description of Student Participants: a. The description of the students who were included in the study is unclear. Please clarify: a)Were all cohort years included? b) Do participants have a specific background related to mental health training? c) How will racial, cultural, and academic diversity be controlled or analyzed? b. It is important to explain whether the curricula at the two universities are similar, given that the results obtained will be compared.

- Explanation has been added to the Ethical Considerations section

Simulation Validation and Development Process. It is also necessary to explain how feedback from committee members was collected and analyzed, and how the results were used to revise the simulation.

- Clarification has now been provided in Phase 1 - Development of the Intervention

Data Integration in Mixed Methods. It is advisable to explicitly explain the data integration strategy based on mixed methods methodological references, such as Creswell & Plano Clark.

- Clarification has now been provided in Phase 3 - Interpretive Description Approach

Reviewer #2: The study is highly relevant to the current needs in healthcare education, particularly in preparing nursing students to provide mental health care for migrant populations. However, the protocol lacks sufficient detail regarding Phase One, specifically the development process of the Immersive Virtual Reality (IVR) simulation. It is important to clearly describe how the validated scenarios will be translated into interactive IVR storylines.

- Additional information has now been added to the Phase 1 - Development of the Intervention

Additionally, more explanation is needed about the nature of the interaction between participants and the virtual patients—such as whether participants will receive feedback during the simulation and what form that feedback will take.

- This is now included in the Phase 2 - One-group Pre- and Post-quasi-experimental section

Furthermore, the duration of each IVR session should be specified, as this is critical for understanding the scope and feasibility of the intervention.

- This is now included in the Phase 2 - One-group Pre- and Post-quasi-experimental
