# Supplementary material for: Immersive virtual reality simulation for undergraduate nursing students: Enhancing mental health care for migrants - A mixed method study protocol
Source: PLoS One. 2025 Sep 25;20(9):e0333434. doi: 10.1371/journal.pone.0333434 (PMC12463210; doi:10.1371/journal.pone.0333434)
Supplement: Attachment — Submitted filename: Response_to_reviewers_auresp_2.docx [file pone.0333434.s003.docx]

Dear author(s), thank you for updated manuscript. Please explain specifically and technically how the Immersive Virtual Reality Simulation are delivered, such as:

- VR device used (what specific VR hardware use, how much the resolution, are there inside-out tracking and wireless mobility?)
  - This information has now been included under Phase 2 - One-group Pre- and Post-quasi-experimental
- Technical procedure (Development Phase; Hardware Setup; and Simulation Run)
  - This information has now been included under Phase 1 - Development of the Intervention and Phase 2 - One-group Pre- and Post-quasi-experimental
- Provide the Content of the Simulation (visual; screen capture as supplement file/include within the manuscript)
  - Figures 1 and 2 have now been uploaded and included.
